# A Biomarker Found in Cadmium Exposed Residents of Thailand by Metabolome Analysis

**DOI:** 10.3390/ijerph110403661

**Published:** 2014-04-02

**Authors:** Dhitiwass Suvagandha, Muneko Nishijo, Witaya Swaddiwudhipong, Ruymon Honda, Morimasa Ohse, Tomiko Kuhara, Hideaki Nakagawa, Werawan Ruangyuttikarn

**Affiliations:** 1 Environmental Science Program, Faculty of Science, Chiang Mai University, Chiang Mai 50200, Thailand; E-Mail: dhitiwass@gmail.com; 2 Department of Public Health, Kanazawa Medical University, Uchinada, Ishikawa 920-0293, Japan; E-Mail: hnakagaw@kanazawa-med.ac.jp; 3 Mae Sot General Hospital, Mae Sot District, Tak 63110, Thailand; E-Mail: swaddi@hotmail.com; 4 Department of Nursing, Kanazawa Medical University, Uchinada, Ishikawa 920-0293, Japan; E-Mail: ryumon@kanazawa-med.ac.jp; 5 Japan Clinical Metabolomics Institute, Kahoku, Ishikawa 929-1174, Japan; E-Mails: jcmi-ohse@heart.ocn.ne.jp (M.O.); jcmi-kuhara@bell.ocn.ne.jp (T.K.); 6 Department of Forensic Medicine, Faculty of Medicine, Chiang Mai University, Chiang Mai 50200, Thailand; E-Mail: ruangyuttikarn@gmail.com

**Keywords:** cadmium, metabolomics, orthogonal partial list square-discrimination analysis (OPLS-DA), urinary citrate, Thailand

## Abstract

First, the urinary metabolic profiling by gas chromatography-mass spectrometry (GC-MS), was performed to compare ten cadmium (Cd) toxicosis cases from a Cd-polluted area in Mae Sot (Thailand) with gender-matched healthy controls. Orthogonal partial list square-discrimination analysis was used to identify new biomarker candidates in highly Cd exposed toxicosis cases with remarkable renal tubular dysfunction. The results of the first step of this study showed that urinary citrate was a negative marker and *myo*-inositol was a positive marker for Cd toxicosis in Thailand. In the second step, we measured urinary citrate in the residents (168 Cd-exposed subjects and 100 controls) and found significantly lower levels of urinary citrate and higher ratios of calcium/citrate and magnesium/citrate, which are risk factors for nephrolithiasis, in highly Cd-exposed residents. Additionally, this inverse association of urinary citrate with urinary Cd was observed after adjustment for age, smoking and renal tubular dysfunction, suggesting a direct effect of Cd on citrate metabolism. These results indicate that urinary citrate is a useful biomarker for the adverse health effects of Cd exposure in a Thai population with a high prevalence of nephrolithiasis.

## 1. Introduction

Itai-itai disease is the most severe form of chronic cadmium (Cd) toxicosis, originally discovered in the Jinzu River basin (Toyama Prefecture, Japan); it is characterized by osteomalacia, severe osteoporosis and renal tubular dysfunction [1]. Studies of itai-itai disease patients and residents of Cd-polluted areas in Japan showed increased low-molecular weight proteins such as α_1_-microglobulin (α_1_-MG), β_2_-microglobulins (β_2_-MG) and retinol binding protein (RBP), enzymuria such as N-acetyl-β-d-glucosamidinase (NAG) and glucosuria due to renal tubular dysfunction [2,3]. Increased amino acids in the urine are also found among Japanese residents of the Cd-polluted areas, and the total amino acids and proline in urine have been used as biomarkers of renal tubular dysfunction [1,2,3].

In 2003, environmental Cd contamination has been discovered in the Mae Sot District of Tak Province, located in the northwestern region of Thailand [4,5]. The source of the contamination is believed to be a zinc-rich area located north of the irrigation creeks where a zinc mine had been operated for more than 20 years [4]. The paddy fields in 12 villages of the Mae Sot District were found to contain markedly elevated Cd, and the urinary Cd levels showed that the Cd body burden was high in these residents [5]. Teeyakasem *et al.* reported that increased Cd levels in the urine were associated with increased levels of renal tubular dysfunction biomarkers such as urinary β_2_-MG, α_1_-MG and NAG [6]. Swaddiwudhipong *et al.* also reported an increased serum creatinine (Cr) and decreased glomerular filtration rate (GFR) associated with increased urinary Cd in the inhabitants with urinary Cd ≥ 5 μg/g Cr [5], which the WHO has suggested to be indicative of Cd contamination [7]. Dose-response relationships of urinary β_2_-MG and NAG with urinary Cd were found, suggesting that renal tubular dysfunction was caused by Cd exposure in this area [8]. Moreover, Nambunmee *et al.* reported increased biomarkers of bone resorption associated with decreased calcium reabsorption, indicating the influence of Cd exposure on bone effects in the Thai population [9]. However, the most characteristic health effect in the present Thai Cd-polluted area is a high prevalence of urinary tract stones related to increased urinary Cd [10,11], a phenomenon that has not been reported in Cd-exposed residents in other countries. 

Recently, metabolomics analysis has been performed to identify the alteration of metabolites in the affected people exposed to environmental chemicals and toxicants, and indicate specific biomarkers for early detection [12]. Urinary metabolomics study is the simultaneous analysis of different classes of metabolite in urine, such as organic acids, amino acid, sugar and sugar alcohol, to be able to compare metabolic profile of exposed cases and controls [13,14]. There are some methods to detect metabolites in urine using different techniques such as gas chromatography-mass spectrometry (GC-MS), liquid chromatography-mass spectrometry (LC-MS), and nuclear magnetic resonance-spectrometry (NMR). Particularly, metabolomics analysis using GC-MS is good at quantification of sugar, organic acids and amino acids in urine [13], which are often observed among residents in Cd polluted area [1,2,3]. 

Therefore, we firstly performed a metabolomics analysis based on GC-MS to identify specific biomarker candidates for Cd toxicosis cases in Thailand. Then, as the next step, we investigated the relationships between the biomarker found by metabolomics and urinary Cd among exposed residents in the Mae Sot area to confirm the association between the level of the detected biomarker and Cd exposure in a Thai population. 

## 2. Experimental Section

### 2.1. Study Subjects

A total of 168 residents (83 men and 85 women; mean age 61.6 years [range: 47–86 years]) living in Cd-polluted Mae Sot in the Tak province were selected from 700 subjects who participated in a health impact survey conducted in 2007 [9,15]. The subjects in the Cd-polluted area consisted of two groups, according to the levels of β_2_-MG and Cd in urine collected in 2007: (1) the Cd toxicosis group (47 men and 37 women) with marked renal tubular dysfunction (β_2_-MG ≥ 1,000 μg/g Cr) and high Cd exposure (urinary Cd ≥ 5 μg/g Cr) and (2) the normal function group (36 men and 48 women) with no clear renal dysfunction (β_2_-MG < 300 μg/g Cr) and low Cd exposure (urinary Cd < 5 μg/g Cr). In 2012, we collected morning urine specimens from these 169 residents, measured the Cd, β_2_-MG and NAG levels in urine, and selected ten typical Cd toxicosis cases (four men and six women) for metabolomics analysis based on the following criteria: Cd ≥ 5 μg/g Cr, β_2_-MG ≥ 1,000 μg/g Cr and NAG ≥ 8 U/g Cr in the urine collected in 2012. 

In 2012, a total of 100 residents (50 men and 50 women) living in a non-polluted area in the same district were recruited as controls. The mean age of control subjects was 61.0 years (range: 43–87 years). The control subjects underwent the same urinary analysis as the exposed subjects; from this group, age- and gender-matched 10 controls were selected for metabolomics analysis based on the following criteria: no history of renal disease or diabetes mellitus, Cd < 2 μg/g Cr, β_2_-MG < 300 μg/g Cr and NAG < 8 U/g Cr in the urine in 2012. The mean values with standard deviation (SD) of age, body size, urinary Cd, renal markers and anemia markers and the prevalence of hypertension, diabetes mellitus and nephrolithiasis for typical Cd toxicosis cases and metabolomics controls are shown in Table 1. No significant differences in age, BMI or prevalence of common diseases were found between the cases and controls, but the levels of Cd, β_2_-MG, NAG and amino acids in the urine, serum Cr and anemia markers for the Cd toxicosis cases were significantly higher than those for the controls.

As the second step after identifying the specific biomarker candidate to Cd toxicosis by metabolomics analysis, the biomarker was measured in urine samples taken in 2012 from all 169 residents in the Cd-polluted area and from the 100 controls in a non-polluted area. The association between the urinary Cd and new biomarker levels was investigated to confirm that the new biomarker is a good indicator of the health effects induced by Cd exposure.

The study protocol was approved by the Research Ethical Committee of the Faculty of Medicine, Chiang Mai University (Approval No. 004/2012). Because the subjects were selected before the survey, we used the data from a previous survey conducted in 2007 by a research team including medical staffs of Mae Sot General Hospital, researchers of Chiang Mai University and Kanazawa Medical University. Before the survey was performed, a medical doctor and medical staffs of Mae Sot General Hospital informed the participants about the study objectives and asked them for their consent to be enrolled in this study. 

**Table 1:** Clinical characteristics of cadmium toxicosis cases and controls in a non-polluted area.

		Control (N = 10)			Cd Toxicosis Cases (N = 10)			
		Mean	SD	Min–Max	Mean	SD	Min–Max	*P*-Value
Age	years	67	8.6	48–77	71	10.6	49–82	NS
Gender ratio	Men/Women	4/6			4/6			
Height	cm	151.6	4.9	143–159	149.5	8.7	137–168	NS
Weight	kg	56.3	6.7	45–65	47.4	11.9	29–65	NS
BMI	kg/m2	23.8	3.1	18.0–28.9	21.3	5.4	12.1–31.8	NS
Urinary Cd ^#^	μg/g Cr	0.87	1.6	0.43–2.1	11.5	1.2	9.2–18.7	***
Urinary β_2_-MG ^#^	μg/g Cr	132	1.7	62–278	33,266	2.3	10,366–138,413	***
Urinary NAG ^#^	U/g Cr	4.1	1.4	2.6–6.5	14.5	1.5	9.5–29.4	***
Urinary amino acids ^#^	μg/g Cr	96	1.3	70.9–171.9	124	1.2	99.6–167.4	*
Urinary proline ^#^	μg/g Cr	4.1	1.4	2.3–6.6	5.8	1.6	2.8–11.5	NS
Serum Cr	mg/dl	0.93	0.18	0.6–1.2	1.62	0.7	1.1–3.4	*
RBC	×10,000	508	44	434–599	406	56	333–514	***
Hb	g/dl	13.5	1.7	11.3–16.3	11.5	1.1	10.2–13.6	**
Ht	%	42.2	4.5	36.4–50.3	35.1	3.2	31.1–41.0	**
Hypertension	N(%)	5	(50)		6	(60)		NS
Diabetes meritus	N(%)	0	(0)		0	( 0)		NS
Nephrolithiasis	N(%)	1	(10)		3	(30)		NS

Note: ^#^: geometrical mean and standard, N: number of subjects, SD: standard deviation, NS: not significant, Min: minimum, max: maximum; BMI: weight (kg)/(Height (m))^2^, Cr: creatinine, N: number of subjects; *: *p* < 0.05, **: *p* < 0.01, ***: *p* < 0.001.

### 2.2. Urine Collection and the Measurement of Renal and Exposure Markers

Approximately 25–30 mL of urine was collected from each subject in the early morning before breakfast. The urine pH was measured immediately using a pH indicator strip (Merck, Darmstadt, Germany) and adjusted to ≥pH 6 to avoid the degradation of β_2_-MG in acidic urine. The urine specimens were transported in a cool container and frozen at −20 °C in the hospital laboratory within 3 hours (average 2.5 h) after collection. To measure renal and exposure markers, the samples were transported to Kanazawa Medical University, Japan in a container with dry ice and kept frozen until analysis.

Urinary Cd was analyzed using a graphite furnace atomic-absorption spectrometer (Shimadzu AA 6300, Kyoto, Japan). The analytical techniques were validated, and the quality assurance of the analysis used urine standard reference material No. 2670 (The National Institute of Standards, Washington, DC, USA) as a quality control. The concentration of urinary β_2_-MG was measured via enzyme immunoassay using a latex agglutination immunoassay (Eiken Chemical, Tokyo, Japan); urinary NAG was measured via a colorimetric assay using the NAG test kit (Shionogi Pharmaceuticals, Osaka, Japan). Urinary calcium (Ca), magnesium (Mg) and phosphorus (P) were determined via colorimetric assays (OCPC for Ca, xylidyl blue method for Mg and phosphomolybdic acid method for P; Clinimate test kit, Sekisui Medical Ltd., Tokyo, Japan) using an automated analyzer (BioMajesty JCA-BM1650, JEOL Ltd., Akishima, Japan). The urinary total amino acids and proline of the typical cases and their age- and gender-matched controls were measured using the colorimetric assay developed by Fukushima *et al.* [16]. All urinary markers were corrected by the urinary Cr concentrations, as determined by the kinetic measurement based on the Jeffe reaction. 

### 2.3. Metabolomics Analysis and Quantification Analysis by GC-MS

To pretreat the samples, the urea in urine was decomposed using a urease treatment with 30 units of urease (Sigma, Alexander City, AL, USA) per 100-μL sample. The 20 stable isotope-labeled compounds (D3-creatinine, D2-glycine, D3-leucine, N2-uracil, D3-methionine, D5-phenylalanine, D4-lysine, N2-orotate, D3-methylcitrate, D4-tyrosine, D4-tyrosine, D4-cistine, D8-homocystine, heptanoylglycine, 2,2-dimethylsuccinate, and 2-hydroxyundecanoate were used as internal standards for the quantification analysis to demonstrate the accuracy of the measurement. After the protein in the urine sample was precipitated, the residue was derivatized by adding 100 μL of N,O-bis-(trimethylsilyl) trifluoroacetamide (BSTFA) with 1% trimethylchlorosilane (TMCS). The metabolites in the derivatized samples were analyzed using GC-MS with a 7890A gas chromatograph and a DB-5MS GC column (Agilent J&W, Santa Clara, CA, USA) coupled with a 5975A inert XL-mass selective detector (MSD; Agilent). The conditions used for GC-MS are described elsewhere [13]. The mass spectra data acquisition system used was GC-MSD Chem Station software (Agilent). We used MetAlign software for data preprocessing of GC-MS raw data. MetAlign offers the data matrix aligned with the retention time (RT) and m/z without missing values by performing peak detection, and the peak alignment to convert peak high to countable data and to adjust all peaks based on Cr peak to make them possible for statistical analysis. The identified compounds, mass spectra and chromatographic retention times were compared with the reference databases of the National Institute of Standards (NIST) 2008 and of Kanazawa Medical University.

### 2.4. Measurements of Detected Biomarker and Minerals in Urine for All Samples on the Second Step of the Study

Because urinary citrate was detected as a specific biomarker candidate for the health effects of Cd pollution in the Thai population, urinary citrate was measured using an enzymatic method (F-kit Citrate, Roche Diagnostics GmbH, Wibringen, Germany) in all subjects living in Cd-polluted and non-polluted areas. The ratios of Ca to citrate and Mg to citrate were calculated and used to determine the risk factors related to urinary stone formation.

### 2.5. Data Analysis

The general characteristic markers of the subjects and the quantification measurements of the metabolites determined with GC-MS were compared between the Cd-exposed and control groups using the paired *t*-test. The difference in the new biomarker and related factors among three Cd exposure categories were compared using one-way ANOVA. The relationships among urinary Cd, renal markers and the candidate metabolite were analyzed using Spearman’s correlation analysis and a linear regression model after adjusting for covariates. These statistical analyses above were performed using Statistical Package SPSS, Version 15 (IBM Inc., New York, NY, USA). To identify a metabolite candidate that could differentiate Cd toxicosis cases from controls, orthogonal partial least squares discriminate analysis (OPLS-DA) was performed using SIMCA-P+ software, Version 12 (Umetrics, Umea, Sweden). Score-plots (S-plots) for the separation of case-control data and variable importance (VIP) for the confirmation of the importance or power of the selected candidates were used to select metabolite candidates. 

## 3. Results and Discussion

### 3.1. Determination of Biomarkers Specific to Cd Exposed Subjects in Thailand

**Figure 1:**
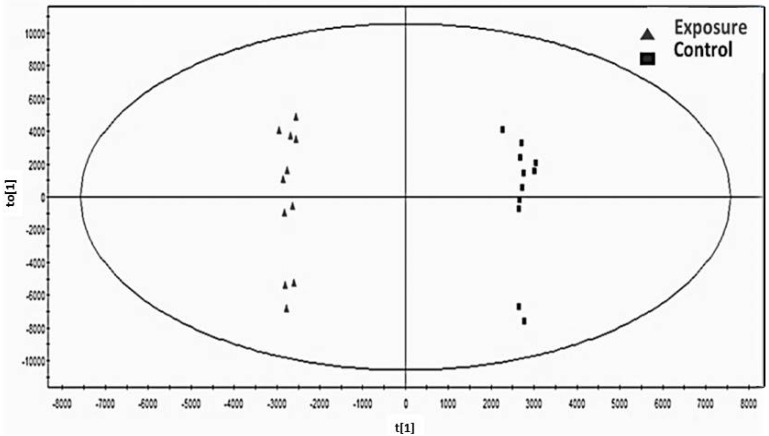
OPLS-DA score plot shows complete discrimination between the Cd exposure group (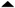) and control group (■) with no variation between two groups.

Figure 1 shows the OPLS-DA score plot derived from the GC-MS spectra indicating the differentiation of the first predictive component (t1) of chromatogram peaks high with RT from 4.09 to 15.00 minutes between the exposed subjects (marked with triangles) and control subjects (marked with squares). Good partitioning between the two groups was observed, and there were no variations among the subjects within each group and no outliers shown by the vertical axis (to 1). These results indicate that this OPLS-DA model fits and is useful for discriminating between these two groups. In the model, the numbers were 19 for observation (N) and 2,523 for variables (k), and model parameters were 0.634 for R2X (cum), 0.996 for R2Y (cum) and 0.840 for Q2 (cum). Significance of the model was confirmed by CV (cross validated goodness of fit)—ANOVA (F = 5.262, *P* = 0.011).

**Figure 2:**
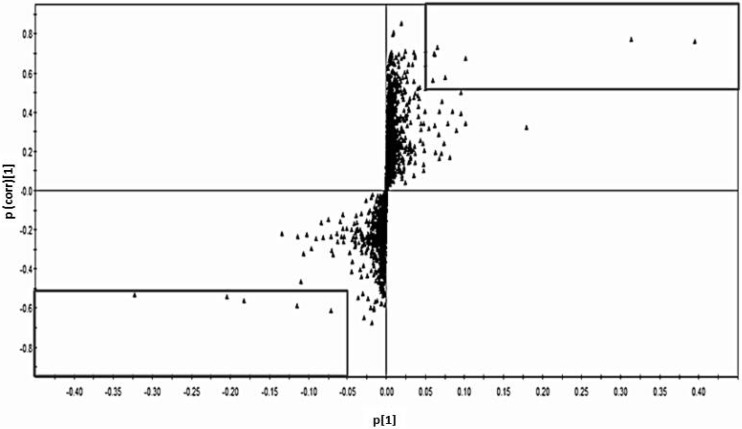
OPLS-DA S-plot of each variable that used a cut-off value for covariance of *p* ≥ |0.05| (magnitude) and p(corr) ≥ |0.5| (reliability), which indicates the most different compounds for each group.

**Figure 3:**
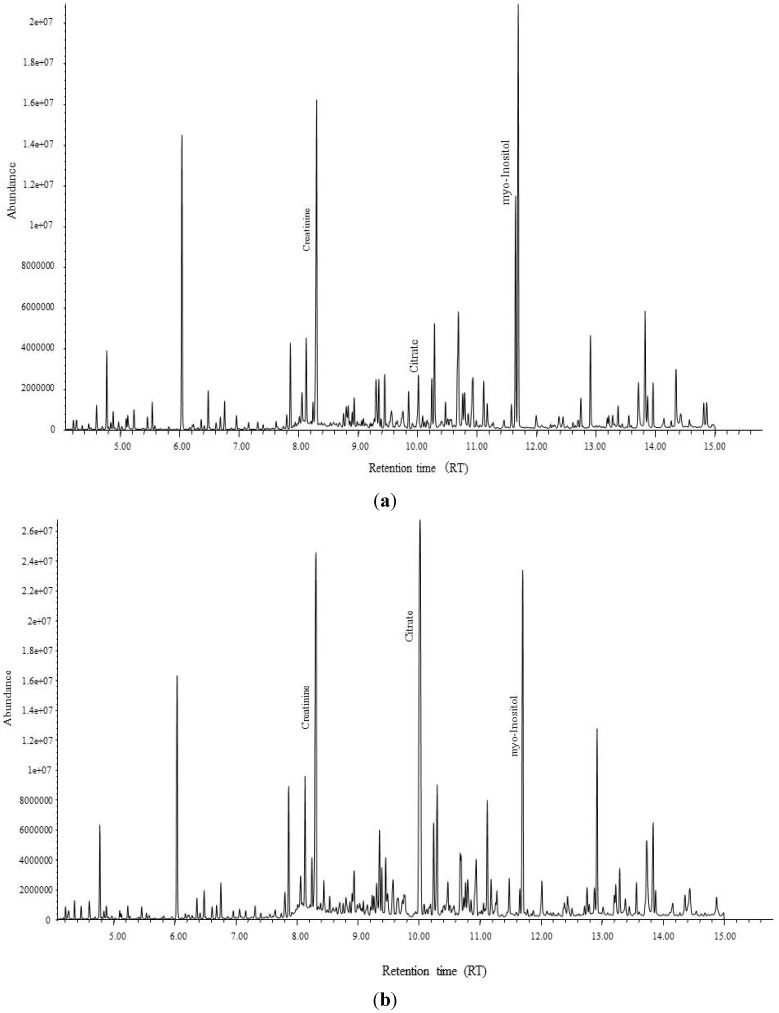
GC-chromatogram of (**a**) a highly Cd exposed case in Mae Sot, and (**b**) a control case in non-polluted area.

To find a metabolite candidate that could discriminate between the exposed and control groups, S-plots from the OPLS-DA model were created using the loading profile of the first component (p) and the correlation of p with the first component (p(corr.)), which represents the reliability of p for the first component (Figure 2). Each plot represents one chromatogram of a compound with a specific RT, and several plots (the compound expressed by its RT) with *P* ≥ 0.05 and p(corr.) ≥ 0.4, located inside the squares in Figure 2, were selected. A compound with RT = 10.02, which was higher in the controls than in the exposed cases (located in the right-side square in Figure 2), and another component with RT = 11.64, which was lower in the controls than in the exposed cases (located in the left-side square in Figure 2), were identified as candidate biomarkers after confirming the VIP values that could discriminate between the two groups. Their VIP values were large enough to confirm their importance for model prediction, with 19.8 for the compound with RT = 10.02, and 10.2 for the compound with RT = 11.64. Based on the RT of the GC/MS spectral database, these compounds were identified as citrate (RT = 10.02) and myo-inositol (RT = 11.64). Chromatograms showing the peaks of citrate and myo-inositol for a Cd toxicosis case and a control are shown in Figure 3.

In addition, for quantification using, internal standards, D3-methylcitrate and 2-hydroxyundecanoate, were used to measure the concentrations of urinary citrate and *myo*-inositol, respectively, and urinary citrate was confirmed to be significantly lower and *myo*-inositol to be significantly higher in Cd toxicosis cases compared with their age- and gender-matched controls (Table 2).

### 3.2. Differences of Present Results in Thai Population from Previous Metabolomics Studies

In this study, we identified urinary citrate and *myo*-inositol as specific biomarker candidates to discriminate Cd toxicosis cases from matched controls using GC-MS-based metabolome analysis in Thai population. In previous studies, several metabolome analyses were performed to identify metabolic biomarkers for the early detection of the nephrotoxicity of drug and environmental toxicants, such as Cd and methyl mercury, in rats using urine and plasma specimens and kidney tissue supernatants [17,18,19,20]. Boudonck *et al.* reported increased polyamines and amino acids in the urine and decreased amino acids and nucleotides in rat kidney tissue exposed to antibiotics and cisplatin before the appearance of histological kidney damage using a combination of GC/MS and LC/MS [18]. Sieber *et al.* compared the urinary metabolomics profiles of rats exposed to ochratoxin A based on GC-MS and H-NMR and found decreased 2-oxisoglutanate and citrate and increased glucose, 5-oxoproline, *myo*-inositol and amino acids in the urine [19]. In rats exposed to other nephrotoxins, such as lithium, similar changes in metabolites were observed in the urine [20]. Two reports discussed Cd-exposed rats, but their results were contradictory; Nicholson *et al.* found decreased urinary citrate in acutely exposed rats [21], whereas Griffin *et al.* reported increased urinary citrate in chronically exposed rats [17].

Regarding humans, we could not find an epidemiological survey of urinary metabolic profiles in residents with long-term exposure to Cd in the environment, with the exception of a study in the United Kingdom (UK) of residents living near a zinc smelter [22]. The authors of that study used high-resolution H-NMR spectroscopy to determine six urinary metabolites, including citrate that correlated with urinary Cd. In addition, they found inverse correlation of urinary citrate with smoking status independent from urinary Cd level [22]. These results are inconsistent with our results in the present study; inverse correlation of urinary Cd and no relationship of smoking status with urinary citrate.

However, the median urinary Cd level of their population in the UK was 0.22 nmol/mmol creatinine (Cr), or approximately 0.22 μg/gCr [23], which was much lower than that in the inhabitants living in the Cd-polluted area in Mae Sot, Thailand. Moreover, the prevalence of higher urinary NAG levels than the reference level (1.25 IU/mmol Cr) was only 9.4% (17 subjects) in residents of UK, although a significant correlation was exhibited between urinary NAG and Cd [23]. Contrary, our present study targeted people including Cd toxicosis cases with remarkable renal tubular dysfunction induced by environmental Cd pollution. Therefore, renal dysfunction with high exposure level might be one reason for the different relationship of urinary citrate with urinary Cd from their study in the UK.

**Table 2:** Comparison of urinary citrate and *myo*-inositol concentrations measured by quantification analysis using GC-MS.

		Controls		Cd toxicosis cases		
		(N = 10)		(N = 10)		
		Mean	SD	Mean	SD	*P*-Value
Urinary citrate	mmol/molCr	3.94	1.42	0.93	2.37	***
Urinary *myo*-inositol	mmol/molCr	0.22	2.51	1.26	3.07	***

Note: Mean: geometrical mean, SD: geometrical standard, ***: *p* < 0.001.

### 3.3. The relationships of Detected Biomarkers and Cd Exposure

Urinary *myo*-inositol, a glucose-alcohol, is known as a renal marker of diabetic nephropathy. In the present subjects, *myo*-inositol seemed to be one of renal markers for detection of renal tubular dysfunction induced by Cd. In our previous report targeted Japanese Cd-exposed population, *myo*-inositol was also detected to be a good biomarker which discriminate sever Cd induced nephropathy cases from gender and age matched controls [24]. We will discuss about *myo*-inositol as a biomarker of Cd nephropathy in another literature.

However, urinary citrate is an organic acid that is a protective factor against urinary stone formation, which is an important health problem in these Mae Sot residents. Therefore, we investigated the relationship between urinary citrate and urinary Cd and other urinary stone-forming factors, such as urinary minerals and the ratios of Ca/citrate and Mg/citrate, in all subjects (including controls) who participated in the survey in 2012. We used one-way ANOVA to compare the urinary concentrations of citrate, Ca, Mg and P and the urinary ratios of Ca/citrate and Mg/citrate among three groups: controls, low-exposure subjects (urinary Cd < 5 μg/gCr), and high-exposure subjects (Cd ≥ 5μg/gCr) of both genders. The results are shown in Table 3. The urinary citrate concentrations in both the low- and high-exposure groups were significantly lower than those in the controls for both men and women. Although no significant differences in the urinary levels of Ca, P and Mg were found in the exposed subjects and controls, the urinary ratios of Ca/citrate and Mg/citrate were significantly higher in the high-exposure subjects than in the controls for both genders. In addition, the ratio of Mg/citrate in the low-exposure women was also significantly higher than that in the controls. These findings suggest that Cd exposure influences citrate metabolism directly and increases the ratios of Ca/citrate and Mg/citrate. These results were inconsistent with study results *in vitro* and *in vivo* animal studies to report that direct Cd exposure at high-level impaired the citrate uptake by the brush border membrane vesicles of renal tubuli, and that the volume of the vesicles was significantly reduced (50%) in exposed rats [25], suggesting increased citrate in urine. However, it is reported that a low extra-vesicular pH stimulates transport citrate in renal brush border membrane vesicles and increased reabsorption of citrate [26], leading to low citrate in urine. Moreover, Tanner *et al* reported that dietary citrate salts improve acidic condition, an alkalinizing effect, and renal function in rats with polycystic kidney [27]. Therefore, Cd may influence the other mechanism than reabsorption on renal tubules such as absorption on intestinal membrane or enzyme activity involved in citrate metabolism. More studies are necessary to clarify the mechanism of decreased citrate related increased Cd exposure. 

In human, the ratios of Ca or Mg/citrate in urine, which are well-known risk factors of urinary stone formation, also increased with increasing urinary Cd levels in these subjects, suggesting that decreased urinary citrate that results in an increased Ca/citrate or Mg/citrate ratio may contribute to the high prevalence of nephrolithiasis in the Thai population in this Cd-polluted area. (see more discussion in Discussion Section 3.5)

**Table 3:** Comparisons of citrate, calcium and magnesium in urine among subjects with different exposure levels, as analyzed by one-way ANOVA.

	Area	Non-Polluted		Polluted				Model	Comparison
		Control		Low exposed		High exposed		ANOVA	between groups
		Mean	SD	Mean	SD	Mean	SD	*P*-value	*P*-value
Men		N = 50		N = 39		N = 44			
	Citrate^#^	0.18	2.18	0.10	2.63	0.05	2.61	0.000	**: C-L, ***: C-H
	Ca^#^	0.05	2.51	0.05	2.37	0.06	2.30	0.523	NS
	P^#^	0.26	1.61	0.23	2.16	0.23	1.72	0.485	NS
	Mg^#^	0.04	1.56	0.04	1.67	0.04	1.68	0.336	NS
	Ca/Citrate	0.54	0.81	0.84	1.29	1.61	1.57	0.000	***: C-H
	Mg/Citrate	0.32	0.32	0.58	0.77	1.28	1.51	0.000	***: C-H
Women		N = 50		N = 47		N = 38			
	Citrate^#^	0.28	2.02	0.13	3.12	0.07	2.93	0.000	***: C-L, ***: C-H
	Ca^#^	0.07	2.28	0.06	2.49	0.08	2.70	0.481	NS
	P^#^	0.34	1.49	0.28	1.63	0.30	1.73	0.176	NS
	Mg^#^	0.04	1.78	0.05	1.59	0.05	1.60	0.211	NS
	Ca/Citrate	0.43	1.01	0.91	1.23	1.79	1.73	0.000	***: C-H
	Mg/Citrate	0.27	0.51	0.72	0.9	1.20	1.01	0.000	*: C-L, ***: C-H

Note: ^#^: Geometrical mean and standard for urinary Cd, citrate, Ca, P and Mg; SD: standard deviation, N = number of subjects, C: controls, L: low exposed group, H: high exposed group; Cr: creatinine, Ca: calcium, Mg: magnesium, Cd: cadmium, β2-MG: beta-2-micloglobuline, NAG: N-acetyl-β-D-glucosamidinase; *: *p* < 0.05, **: *p* < 0.01, ***: *p* < 0.001.

### 3.4. The Relationships of Detected Biomarkers and Renal Effects

Moreover, urinary citrate and the ratios of Ca/citrate and Mg/citrate were significantly correlated with both Cd and urinary β_2_-MG for both genders (Table 4). However, urinary NAG was significantly correlated only with Mg/citrate ratio in women, suggesting that Cd exposure affect citrate metabolism directly and increased urinary β_2_-MG through other than renal tubular dysfunction, as indicated by the increased NAG. To confirm the association between urinary Cd and urinary citrate after adjusting for age, smoking status and urinary β_2_-MG and NAG levels, multiple linear regression analysis with three types of models was performed for all of the participants in the 2012 survey. Table 5 shows that significantly decreased levels of urinary citrate, and increased ratios of Ca/citrate and Mg/citrate in proportion to increasing urinary Cd were found after adjustment for only age and smoking (Model 1) or age, smoking and NAG (Model 3) for both genders. 

**Table 4:** Correlation coefficients (Spearman’s ρ) of citrate, calcium and magnesium in urine with Cd and renal tubular markers among the subjects participated in 2012 survey.

		Citrate		Ca		P		Mg		Ca/Citrate		Mg/Citrate	
Men (N = 133)													
	Cd	−0.482	***	0.067		−0.122		0.084		0.448	***	0.463	***
	β2-MG	−0.323	***	−0.034		−0.128		0.182	*	0.275	**	0.360	***
	NAG	−0.112		−0.175	*	−0.034		0.057		0.038		0.115	
Women (N = 135)													
	Cd	−0.504	***	0.151		−0.089		0.108		0.574	***	0.554	***
	β2-MG	−0.497	***	−0.011		−0.030		0.058		0.465	***	0.499	***
	NAG	−0.159		0.015		−0.033		0.116		0.160		0.129	*

Notes: Urinary Cd, citrate, Ca, P and Mg were corrected by urinary creatinine, and transformed to logarismic values. Cr: creatinine, Ca: calcium, Mg: magnesium, N: number of subjects, Cd: cadmium, β2-MG: beta-2-micloglobuline, NAG: N-acetyl-β-d-glucosamidinase, *: *p* < 0.05, **: *p* < 0.01, ***: *p* < 0.001.

**Table 5:** Standardized regression coefficients (β) for urinary cadmium and renal markers in linear regression model for urinary citrate, calcium/citrate and magnesium/citrate.

	Explanatory Factors	Citrate		Ca/Ctrate		Mg/Ctrate	
		β	*P*−Value	β	*P*−Value	β	*P*−Value
***Men***	(N = 133)						
Model 1	Age	−0.050		−0.119		0.031	
	Smoking	0.030		−0.311		−0.130	
	Cd	−0.451	***	0.311	***	0.349	***
Model 2	Age	0.004		−0.200	*	−0.042	
	Smoking	0.018		−0.110		−0.114	
	Cd	−0.373	***	0.193		0.243	*
	β2-MG	−0.155		0.233	*	0.209	
Model 3	Age	−0.058		−0.112		−0.025	
	Smoking	0.028		−0.126		−0.142	
	Cd	−0.458	***	0.316	**	0.302	**
	NAG	0.024		−0.021		0.166	
***Women***	(N = 135)						
Model 1	Age	−0.152		0.070		0.220	**
	Smoking	−0.107		0.213		0.116	
	Cd	−0.445	***	0.329	***	0.341	***
Model 2	Age	0.021		−0.043		0.077	
	Smoking	−0.084		0.197	*	0.096	
	Cd	−0.271	**	0.215	*	0.196	*
	β2-MG	−0.382	***	0.250	*	0.317	**
Model 3	Age	−0.213	*	0.113		0.269	**
	Smoking	−0.122		0.224	**	0.129	
	Cd	−0.485	***	0.358	***	0.373	***
	NAG	0.152		−0.109		−0.122	

Note: Urinary Cd, citrate, Ca, P and Mg were corrected by urinary creatinine, and transformed to logarismic values, Cr: creatinine, Ca: calcium, Mg: magnesium, Cd: cadmium, β2-MG: beta-2-micloglobuline, NAG: N-acetyl-β-d-glucosamidinase, *: *p* < 0.05, **: *p* < 0.01, ***: *p* < 0.001.

In women, urinary citrate, Ca/citrate and Mg/citrate were independently related to both urinary Cd and β_2_-MG after adjusting for age and smoking (Model 2). In men, however, urinary citrate and Mg/citrate were significantly related to urinary Cd independent of age, smoking and urinary β_2_-MG, and Ca/citrate was related to both age and β_2_-MG but not to urinary Cd due to multi-co-linearity between urinary Cd and β_2_-MG (Model 2). In the non-smokers (55 men and 112 women), same analysis with three models was performed, but the relationships adjusted by age between three citrate markers and urinary Cd and renal markers were similar to those in all participants (see details in Appendix). Because Cd exposure induces renal tubular dysfunction, renal tubular dysfunction may cause alterations in urinary citrate excretion. However, urinary NAG was not associated with urinary citrate with or without adjusting for age and urinary Cd for both genders. Although urinary β2-MG is a good marker for renal tubular dysfunction, urinary β2-MG has been reported to increase in renal stone formers in non-exposed population [28]. Therefore, increasing urinary β2-MG may be caused by not only Cd induced renal tubular dysfunction but also by decreased urinary citrate in Cd exposed subjects. These results suggest that Cd exposure has a direct effect on citrate metabolism that is secondary impaired renal tubular dysfunction. In animal study, Gadola et al. reported that calcium citrate slows the progression of chronic renal injury in the rats with 4/5 nephrectomy of renal failure model [29].

### 3.5. Cd Pollution and Nephrolithiasis

Increased urinary stone formation was previously reported in Swedish factory workers [30,31], but there is no evidence of an increased prevalence of nephrolithiasis in Japanese and Belgian Cd-polluted areas. Only in the present Thai Cd-polluted area has the high prevalence of urinary calculus related to increasing urinary Cd been reported in Cd-exposed subjects [10,11]. In the present study, prevalence of urinary calculus obtained by interview was 4.3% (three men and four women) in the polluted area and 1% (only one man) in a non-polluted area, although the difference was not significant. In comparison, in non-polluted areas, the high prevalence of renal stones with hypocitraturia related to renal tubular acidosis caused by K deficiency has been reported in northern Thailand [32,33]. This pre-existing condition might enhance the effect of Cd on citrate metabolism and increase the prevalence of renal stones in the Mae Sot area, which is located in northern Thailand. Therefore, the present discovery of the citrate metabolism alterations caused by Cd exposure will aid the early detection, prevention and treatment of renal stones, which are a known health problem in the Cd-exposed Thai population. In future, a bigger scale epidemiological survey including dietary habit and epigenetic factors will be needed to investigate Cd effects on citrate metabolism and renal dysfunction in Mae Sot residents in Thailand.

## 4. Conclusions

The present study indicated that urinary citrate and myo-inositol may be useful biomarker candidates in Cd-exposed subjects in the Thai population. Additionally, the association of urinary citrate with Cd exposure was confirmed in all residents living in Mae Sot, Thailand, and the usefulness of urinary citrate was suggested for the early detection and prevention of nephrolithiasis.

## Appendix

**Table A1:** Standardized regression coefficients (β) for urinary cadmium and renal markers in linear regression model for urinary citrate, calcium/citrate and magnesium/citrate in non-smokers.

	Explanatory Factors	Citrate		Ca/Ctrate		Mg/Ctrate	
		β	*P*-Value	β	*P*-Value	β	*P*-Value
***Men***	*(N = 55)*						
Model 1	Age	−0.010		−0.075		0.014	
	Cd	−0.555	***	0.341	*	0.381	**
Model 2	Age	0.110		−0.200	*	−0.042	
	Cd	−0.388	**	0.193		0.243	*
	β2−MG	−0.310		0.233	*	0.209	
Model 3	Age	−0.009		−0.037		−0.047	
	Cd	−0.555	***	0.380	*	0.320	*
	NAG	0.003		−0.119		0.188	
***Women***	*(N = 112)*						
Model 1	Age	−0.178		0.145		0.232	**
	Cd	−0.427	***	0.264	**	0.286	***
Model 2	Age	−0.021		0.004		0.080	
	Cd	−0.285	**	0.136		0.148	
	β2−MG	−0.321	**	0.288	*	0.312	*
Model 3	Age	−0.269	**	0.247	*	0.307	**
	Cd	−0.468	***	0.310	**	0.320	**
	NAG	0.199	*	−0.223	*	−0.163	

Note: Urinary Cd, citrate, Ca, P and Mg were corrected by urinary creatinine, and transformed to logarismic values, Cr: creatinine, Ca: calcium, Mg: magnesium, Cd: cadmium, β2-MG: beta-2-micloglobuline, NAG: N-acetyl-β-D-glucosamidinase, *: p < 0.05, **: p < 0.01, ***: p < 0.001.
